# Stroke genomics in people of African ancestry: charting new paths

**DOI:** 10.5830/CVJA-2015-039

**Published:** 2015

**Authors:** RO Akinyemi, B Ovbiagele, M Gebreziabher, S Warth, D Lackland, A Akpalu, K Sagoe, L Owolabi, F Sarfo, R Obiako, H Tiwari, D Arnett, RO Akinyemi, RN Kalaria, E Melikam, O Arulogun, A Ogunniyi, MO Owolabi

**Affiliations:** Division of Neurology, Federal Medical Centre Abeokuta, Nigeria; Department of Neurosciences, Medical University of South Carolina, USA; Department of Neurosciences, Medical University of South Carolina, USA; Department of Neurosciences, Medical University of South Carolina, USA; Department of Neurosciences, Medical University of South Carolina, USA; College of Health Sciences, University of Ghana, Accra, Ghana; College of Health Sciences, University of Ghana, Accra, Ghana; Department of Medicine, Bayero University, Kano, Nigeria; School of Medical Sciences, Kwame Nkrumah University of Science and Technology, Kumasi, Ghana; Department of Medicine, Ahmadu Bello University, Zaria, Nigeria; Department of Public Health, University of Alabama at Birmingham, USA; Department of Public Health, University of Alabama at Birmingham, USA; Institute of Neuroscience, Newcastle University, UK; Institute of Neuroscience, Newcastle University, UK; College of Medicine, University of Ibadan, Ibadan, Nigeria; College of Medicine, University of Ibadan, Ibadan, Nigeria; College of Medicine, University of Ibadan, Ibadan, Nigeria; College of Medicine, University of Ibadan, Ibadan, Nigeria

**Keywords:** stroke, cerebrovascular risk factors, genomics, genetics, Nigeria, Ghana, Africa, African ancestry

## Abstract

One in six people worldwide will experience a stroke in his/her lifetime. While people in Africa carry a disproportionately higher burden of poor stroke outcomes, compared to the rest of the world, the exact contribution of genomic factors to this disparity is unknown. Despite noteworthy research into stroke genomics, studies exploring the genetic contribution to stroke among populations of African ancestry in the United States are few. Furthermore, genomics data in populations living in Africa are lacking. The wide genomic variation of African populations offers a unique opportunity to identify genomic variants with causal relationships to stroke across different ethnic groups. The Stroke Investigative Research and Educational Network (SIREN), a component of the Human Health and Heredity in Africa (H3Africa) Consortium, aims to explore genomic and environmental risk factors for stroke in populations of African ancestry in West Africa and the United States. In this article, we review the literature on the genomics of stroke with particular emphasis on populations of African origin.

## Abstract

Stroke is the clinical culmination of several complex processes and interacting pathways that involve various genetic and environmental factors.^1^ However, the exact nature and level of the contribution of genetic factors to stroke and its different subtypes have not been clearly established. Presumably, genetic contributions to stroke may result from common variants with small effect sizes, rare variants with large effect sizes, or a combination of both.^2^-^4^ Nevertheless, studies exploring the genetic underpinnings of the peculiarities of stroke in populations of African ancestry in the United States are few,^3^,^5^-^7^ while there are hardly any data on populations living in Africa.

The diverse genomic variation of African populations^8^-^10^ offers a unique opportunity to identify novel genes and molecular pathways of stroke that may lead to new and better prevention and treatment options for stroke in people of African ancestry and other global populations. Understanding the interplay of genetic and environmental risk factors for stroke is critical to the prediction of its occurrence, severity and outcome as well as the formulation of successful tailored treatment and prevention programmes. In addition, the biology of stroke subtypes will be better deciphered.

In this review article, we provide an overview of the changing global and in particular, African epidemiology of stroke, the known peculiarities of stroke in Africa, extant literature on the genomics of stroke and cerebrovascular risk factors, with particular attention to people of African ancestry, as well as opportunities for charting new paths through the Human, Health and Heredity in Africa (H3Africa) initiative.^11^,^12^

## Changing global and African epidemiology of stroke

Stroke has attracted global attention, as one in six people will develop stroke in their lifetime.^13^,^14^ Stroke is a significant medical and public health problem, with loss of productivity and burden on families, caregivers and society.^13^-^15^ The lifetime risk of stroke is one in five women and one in six men.^13^ Stroke is the most common cause of acquired disability and the second most common cause of death worldwide.. The World Health Organisation (WHO) estimates for 2001 indicated that death from stroke and disability-adjusted life years (DALYs) due to stroke was at least seven times higher in low- and middle-income countries (LMIC) than in high-income countries (HIC).^16^ Recent incidence estimates^17^,^18^ indicate that whereas stroke incidence declined 12% in HIC, it increased by 12% in LMIC over the last decade.

Africa, in epidemiological transition,^19^,^20^ is currently faced with an exploding but neglected burden of non-communicable diseases (NCDs), including hypertension, diabetes mellitus and dyslipidaemia, which often culminate in stroke. The recent United Nations high-level meeting on the global burden of NCDs highlighted their disproportionately high burden and stressed the urgent need to tackle them, particularly in developing countries.

The burgeoning incidence of stroke in Africa is attributable to rising cardiovascular risk profile, which is in turn driven by epidemiological transition, an aging population, rapid urbanisation and accompanying lifestyle changes. Africa, with a current population of over one billion, has a stroke prevalence rate of up to 963/100 000 population,^21^ an incidence rate of up to 315/100 000 population and a three-year mortality rate as high as 84%. About 3.2 million Africans develop incident stroke every year.^15^ Recent data from Nigeria, Tanzania and Sudan showed that stroke was the leading cause of elderly medical admissions,^22^ while up to 78% of neurological hospital admissions were due to stroke.^23^ The impact of this on mental capacity, quality of life and economic productivity portends great danger for the emerging economies of Africa.^24^-^26^

## Peculiarities of stroke in people of African ancestry

Enhanced predisposition, different pattern of subtypes, worse severity and often poorer outcome of stroke in people of African descent is quite well established. According to data from the INTERSTROKE study, ischaemic stroke accounts for 66% while haemorrhagic stroke accounts for 34% in Africa, compared to 91 and 9%, respectively for ischaemic and haemorrhagic stroke in HIC. Ischaemic stroke subtypes diagnosed in African populations were small vessel (27%), cardio-embolism (25%), large vessel (14%), others (20%) and undetermined (14%).^27^

Among sub-Saharan Africans, stroke affects a relatively younger age group and productive workforce than in developed economies.^27^,^28^ Data from the USA suggest that African Americans have a higher predisposition, worse severity and often poorer outcomes compared to Caucasian Americans.^29^,^30^ In a recent report from the multi-ethnic South London Stroke Registry study, black stroke survivors had worse cognitive outcome compared to other racial groups.^31^ Although, this may be due to socio-economic differences, disparities in healthcare-seeking practices and differential access to healthcare services, the influence of underlying differences in genetic factors cannot be underestimated.^32^,^33^

## Genomics and health disparities

Genetics and genomics research offer insight into disparities in the risk profile, phenotypes and outcome of diseases among different populations as a result of accumulated small differences in common alleles or rare variants, interactions among multiple genetic loci and interactions between genes and environmental factors, which may include cultural practices and health-seeking behaviour.^34^,^35^ The potential of treatment approaches tailored to individual, unique genomic profiles represents a distinct potential impact of genomics on improving health disparities. Also, the globalisation of complex chronic diseases further suggests that all populations are susceptible, and that variation in rates may also be explained as a result of differential exposure to environmental causes, including lifestyles, cultural practices and health-seeking behaviours.^36^

## African human genomic variation

African populations present the highest genomic diversity, the lowest levels and most divergent patterns of linkage disequilibrium, as well as smaller haplotype block sizes across human populations.8,37 Although the human species is believed to have originated from Africa about 200 000 years ago, studies of genomic variation in Africa suggest that the present pattern of variation within and between populations is a product of several factors. These include demographic history, population structure, diversities of geographical location, language classification and different patterns of subsistence, dietary differences, multiple migrations with accompanying high levels of genetic admixture and survival related to exposure to infectious diseases.38,39

For example, Tishkoff and colleagues^8^ identified 14 ancestral population clusters in Africa with four predominant clusters that broadly represent populations from major African geographical regions and the four dominant African language families. These are Niger-Kordofanian (spoken primarily by agriculturalist populations located in large contiguous regions of sub-Saharan Africa from West Africa to eastern and southern Africa), Nilo-Saharan (spoken predominantly by pastoralist populations in central and eastern Africa), Afro-Asiatic (spoken predominantly by agro-pastoralists and pastoralist populations in northern and eastern Africa), and Khoisan (a language family that contains click consonants, spoken by hunter–gatherer San populations in southern Africa as well as the Hadza and Sandawe hunter–gatherers in Tanzania). The remaining 10 are mainly restricted to specific geographic regions, languages, or in some cases, individual populations.

More recently, Shriner and colleagues^9^ analysed ancestry data from 12 global and regional diversity projects with genome-wide genotype data for 3 528 unrelated individuals from 163 samples from around the world. They identified 19 ancestral components with 94.4% of individuals showing mixed ancestry. Furthermore, they validated the earlier findings of Tishkoff and colleagues and identified an additional ancestral component in Africa, the Omotic-speaking peoples of Ethiopia.

Our knowledge of African human genomic variation is growing. This was previously limited by the small number of African populations involved in landmark projects such as the International HapMap project^40^ and the more recent 1 000 Genomes project.^41^ In these projects, participation was limited to largely Niger-Kordofanian-speaking Yoruba and Esan from Nigeria, Mende from Sierra Leone, Bantu-speaking Luhya from Kenya, and Nilo-Saharan-speaking Maasai from Kenya. A large proportion of African human genomic variation therefore remained unexplored.

However, recent data from the African Genome Variation project (AGVP) have provided evidence and more detailed characterisation of African genomic diversity.^10^ The AGVP utilised dense genotypes from 1 481 individuals and wholegenome sequences from 320 individuals across sub-Saharan Africa. Novel evidence of complex, regionally distinct widespread hunter–gatherer and Eurasian admixture across sub-Saharan Africa was apparent and substantial hunter–gatherer and Eurasian ancestry admixture of up to 23 and 50%, respectively, were found in many African populations with detailed chronology of the timing of the admixture. For instance, whereas the Eurasian admixture among the Yoruba occurred 7 500–10 500 years ago, it was more recent among the Fula tribe of Gambia, occurring only about 320–780 years ago.

These admixtures provide evidence for back-to-Africa migration, the existence of hunter–gatherer populations in West Africa and a pattern of gene flow consistent with the Bantu expansion. The AGVP also found new loci related to susceptibility, pathogenesis, severity and outcome of several diseases, including malaria, Lassa fever, trypanosomiasis, trachoma and hypertension. For instance, they identified highly differentiated variants within genes involved in osmoregulation (*ATP1A1* and *AQP2*), deregulation of *AQP2* expression, and loss-of-function mutations in *ATP1A1* have been associated with essential and secondary hypertension, respectively.^42^,^43^ The study also established an efficient genotype array design capturing common genetic variation in Africa, which would be useful for future African genomic studies.^11^,^12^

## African American genomic variation

African Americans have mixed ancestry originating from Africa and other continents, especially Europe. Studies have shown the average amount of African ancestry in African Americans to be about 80% (predominantly of western and central African origin),^8^,^44^,^45^ although there is substantial variation in the level of African ancestry in individual African Americans, as the proportion of African ancestry in a given individual can range from one to 99%.^39^

Genomic variation and related phenotype data on variable traits contribute novel information useful for identifying population-specific variants that play a role in gene function, phenotypic adaptation and susceptibility to complex diseases, such as stroke in Africans and populations of African descent. The APOE ε4 allele, a well-studied example that contributes to a small extent to individual and population risks of traits such as stroke, heart disease and dementia, is found in virtually all populations, albeit at varying rates. The frequency of homo- or heterogeneous APO ε4 alleles varies across populations but confers different attributable risks of Alzheimer’s disease; the risk being higher among the Japanese but much lower among people of African ancestry with higher allele frequencies. This suggests possible intervening roles for epigenetic interactions from certain modifier genes or some other environmental factors.^34^

## Genomics of stroke and cerebrovascular risk factors

Stroke is a complex polygenic, heterogeneous and multifactorial disorder involving many complex mechanisms, intermediate phenotypes and the interplay of genetic and non-genetic factors. Evidence from twin studies, family history studies, animal models and heritability studies of vascular risk factors and intermediate phenotypes suggests a likely significant contribution of genetic factors to the neurobiology and phenomenology of stroke.^1^,^4^,^46^

## Family history and heritability

Among individuals with a positive family history of stroke, there is an increased risk of stroke, which may be due to expression of genetic susceptibility, a shared environment or both.^47^ In the Family Heart study, personal and familial histories of stroke were assessed in 3 168 individuals (probands) who were at least 45 years old and 29 325 of their first-degree relatives. The odds of stroke were 2.00 (1.13–3.54) for a positive paternal and 1.41 (0.80–2.50) for a positive maternal history of stroke after adjusting for age, gender, ethnicity and presence of vascular risk factors, and the pattern was similar between African Americans and European Americans.^48^

In a systematic review of the heritability of stroke in 53 independent studies (three twin studies, 33 case–control studies and 17 cohort studies), it was found that monozygotic twins were more likely to be concordant than dizygotic twins (OR, 1.65; 95% CI, 1.2–2.3; *p* = 0.003) while a positive family history was a risk factor for stroke in both case–control (OR, 1.76; 95% CI, 1.7–1.9; *p* < 0.00001) and cohort (OR, 1.30; 95% CI, 1.2–1.5; *p* < 0.00001) studies. Besides, positive family history was more associated with small-vessel and large-vessel strokes.^49^

## Cerebrovascular risk factors

Genomic factors may contribute to the neurobiology of stroke through their influence on established risk factors, such as hypertension, diabetes, dyslipidaemia, obesity and cigarette smoking or through their influence on intermediate phenotypes, such as white matter hyperintensities (WMH) and carotid intima–media thickness (CIMT). For instance, research evidence has shown racial and ethnic disparities in cardiovascular and cerebrovascular diseases, with Americans of African ancestry showing a higher prevalence of hypertension and earlier onset, and faster and more severe end-organ damage, including stroke.^50^ Apart from non-inherited factors such as lifestyles, health-related practices, socio-economic profile and differential access to healthcare, genetic factors contributed significantly to this disparity.^32^

A recent genome-wide association study (GWAS) of hypertension and blood pressure in African Americans using the pathway-focused approach established the genome-wide significant association of the genetic variants *PMS1, SLC24A4, YWHA7, IPO7* and *CACANA1H* with systolic blood pressure levels, with significant replication of some single-nucleotide polymorphisms (SNPs) in a sample of West Africans.^51^ Using a similar approach, a more recent study has found association between multiple variants in several genes in the adrenergic alpha-1 receptor (ADRA1) pathway and hypertension in Yoruba Nigerians.^52^ A meta-analysis of genome-wide linkage scans for blood pressure variation in Nigerians and African Americans reported association in two loci: 2p14 –p13.1 and 7p21.3 –p15.3, the second locus being attributed to the Nigerian sample and suggesting a unique locus for blood pressure variation in people of African ancestry.^53^

In the GenHAT study evaluating the pharmacogenetic effects of candidate gene complexes on stroke, significant genetic difference was found between hypertension drug treatment groups in patients who had experienced stroke, especially among African Americans and non-Hispanic whites.^54^ Given the fact that hypertension is the most dominant risk factor for stroke among people of African ancestry in Africa,^15^,^24^,^27^,^28^ and the diaspora,^30^,^50^ it would be worthwhile exploring the possible contribution of these hypertension-related genotypes in people of African ancestry.

A Nigerian study assessed glucose and insulin responses to an oral glucose load among offspring of parents with type 2 diabetes mellitus (T2DM) and found higher levels of fasting plasma glucose, fasting plasma insulin, and two-hour post-glucose load plasma insulin, indicating a higher risk for developing diabetes.^55^ A Cameroonian case–control pedigree study showed increased prevalence of diabetes and impaired glucose tolerance in the offspring of parents with T2DM.^56^

The Africa America Diabetes Mellitus (AADM) study has utilised genome-wide linkage and association studies to provide insight into the genomics of T2DM in Niger-Kordofanian African populations of Nigeria and Ghana. Multiple linkage analysis provided evidence of regions of chromosome 12, 19 and 20 (the strongest being 20q13.3).^57^ The loci found to influence C-peptide plasma levels (10q23, 4p15) were found to harbour multiple T2DM candidate genes [phosphatase and tensin homolog (PTEN), protein phosphatase 1, regulatory subunit 3C (PPP1R3C), insulin degrading enzyme (IDE), and peroxisome proliferator activated receptor gamma, coactivator 1 alpha (PPARGC1)].^58^ Collaborative GWAS and other studies have identified further susceptibility (CDKAL1, CAPN10, TCF7L2 variants and PPARG variants) and protective loci (TCF2, AGRP -38C/T).^57^

Chronic kidney disease (CKD) is an identified risk factor for cerebral vascular disease.^59^ Multiple common SNPs in the gene that encodes non-muscle myosin heavy-chain type II isoform A (MYH9) have been associated with an increase in the risk of focal segmental glomerulosclerosis and end-stage renal disease,^60^ while more recently the apolipoprotein L1 (*APOL1*) gene has been identified as a risk locus for CKD in African Americans, and replications confirmed in Nigerian Yoruba CKD patients.^61^,^62^

In Africa, *APOL1* confers resistance to infection from *Trypanosoma brucei brucei*, one of the trypanosomes that cause African sleeping sickness and it is believed that its evolutionary history lies in its positive selection due to its protection against sleeping sickness.^63^ Interestingly, an inverse relationship between high-density lipoprotein cholesterol (HDL-C) and kidney function in African ancestry populations has also been described in individuals with the nephropathy risk *APOL1* gene.^64^ Higher HDL-C was associated with worse kidney function in those with the risk genotype, while no association was observed among those without the genotype. Therefore, the increasing incidence of cardiovascular disorders (CVDs) in Africa along with the evidence of genetic variants that increase susceptibility to CVDs signals the need for large-scale genomic epidemiology studies in Africa in search of other putative protective and susceptibility loci.^12^

Population-attributable risks of genetic variants differ depending on whether they are monogenic, common variants or rare variants of multiple genes of polygenic disorders.^2^,^3^ More important, however, is the functional significance of the variants in the biological pathways where their gene products contribute to the biology of the disease and may possibly be of therapeutic or preventative importance.^65^ This is the major thrust of our proposed study of genetic variants relevant to stroke in people of African ancestry. African representation in the 1 000 Genomes study is limited,^41^ while the H3Africa projects^11^ offer robust opportunities for detailed exploration of genomic data relevant to African and global populations.

## Intermediate phenotypes: WMH and CIMT

Both twin and family studies have shown that magnetic resonance imaging of white matter hyperintensities has shown a heritability (proportion of variation explained by genetic factors) of up to 70%.^66^ CIMT measured by ultrasound and believed to represent the early stages of atherosclerosis and related to large-artery stroke has been estimated to have a heritability of between 30 and 70%.^67^

## Monogenic stroke disorders

Monogenic disorders may cause stroke as part of multi-systemic manifestations or solely as a clinical phenotype limited to the central nervous system. They are important for individual patients but may not account for much population-attributable risk.^46^

Sickle cell disease (SCD) is of particular importance in people of African ancestry. It is caused by a point mutation at codon 6 of the beta-globin gene, leading to a glutamic acid to valine (Glu→Val) substitution in the beta-globin chain of human adult haemoglobin, and producing sickle haemoglobin (HbS). Inherited autosomal recessively, either two copies of HbS or one copy of HbS plus another beta-globin variant (such as HbC) are required for disease expression. HbS carriers are protected from malaria infection, and this selective pressure is believed to have led to the high frequency of HbS (up to 40%) in individuals of African ancestry, especially in areas of high malaria endemicity.^68^,^69^

The spread of SCD to the Americas is inextricably linked to slavery and the large-scale forced translocation of populations from West Africa.^70^ Large- or small-vessel cerebral ‘vasculopathy’ characterised by proximal intracranial arterial stenoses, often leading to a moyamoya pattern, commonly complicates SCD and may manifest as abnormal transcranial Doppler velocity (> 200 cm/s) or frank stroke, particularly in younger patients with sickle cell anaemia, while complicated (hemiplegic) migraine was previously reported in Nigerian adults with sickle cell trait (HbAS).^71^ By middle age, up to 25% of SCD patients develop overt stroke.^72^

Certain genetic polymorphisms may be associated with stroke in SCD as modifier genes. For instance whereas α-thalassaemia genes may be protective, mutations in the glucose-6-phosphate dehydrogenase (G6PD) genes and certain SNPs, including *ANXA2, rs11853426, TEK rs489347*, and *TGFBR3 rs284875* variants, have been associated with increased stroke risk.^73^ A recent whole-exome sequencing (WES) study identified two modifier mutations *GOLGB1 (Y1212C)* and *ENPP1 (K173Q)* associated with protection from stroke in a cohort of children with sickle cell anaemia.^74^

However, the interactions between SCD, its associated modifier genes, and environmental factors to produce an intermediate phenotype (TCD velocity > 200 cm/s) and stroke have not been examined in people of indigenous sub-Saharan Africa. Knowledge of these interactions and the metabolic pathways involved may unmask targets for preventative and therapeutic interventions in the sub-population of people living with SCD.

Cerebral autosomal dominant arteriopathy with subcortical infarcts and leukoencephalopathy (CADASIL) is associated with mutations in the *NOTCH3* gene and presents with migraine headache, followed by depression and ischaemic stroke in the deep gray structures and subcortical white matter, cognitive decline, and dementia.^75^,^76^ A model of small-vessel disease, the first case of CADASIL in populations of African ancestry, was recently reported in a 73-year-old African American with a 15-base-pair heterozygous duplication of the exon 7 of the *NOTCH 3* gene.^77^ Other related monogenic small-vessel cerebrovascular disorders include cerebral autosomal recessive arteriopathy with subcortical infarcts and leukoencephalopathy (CARASIL), retinal vasculopathy with cerebral leukodystrophy (RVCL), and Fabry disease.^78^

## Genetic linkage studies

Genetic linkage studies have contributed to our understanding of the heritability of stroke and especially chromosomal regions and sub-regions involved, even though most studies have focused more on the ischaemic phenotype. Table 1 shows findings from a few linkage studies in stroke,^79^-^82^ including genome-wide linkage studies. Much of these findings are further confirmed by more specific candidate gene analysis and the more rigorous approaches of association studies. A relative strength of linkage studies is the feasibility of working with a few hundred subjects using the case–control approach.

**Table 1:** Genetic linkage studies in stroke

*First author (year)*	*Study type*	*Phenotype*	*Sample*	*Salient findings*
Craig *et al.* (1998)^74^	Linkage analysis	Cerebral cavernous malformation	20 non-Hispanic Caucasian families	CCM – 1(7q) (found in Hispanic Americans), CCM2 (7p13-15) and CCM3 at 3q25.2-27 all found in non-Hispanic Caucasian families.
Nilsson-Ardnor *et al.* (2007)^76^	Genome-wide linkage analysis	All strokes; ischaemic stroke	56 Swedish families with familial stroke	LOD scores > 1.2 at 9 1ocations: 1p34, 5q13, 7q35, 9q22, 9q34, 13q32, 14q32, 18p11, and moderate linkage on chromosomes 5q, 9q, 13q, and 18p.
			Additional 53 families with familial strokes	Analysis of 53 additional families, further confirmed linkage on chromosomes 5q, 13q, and 18p.
Janunger *et al.* (2009)^75^	Genome-wide linkage analysis	All strokes	7 nuclear Swedish families with a common ancestor and connected over 8 generations	A maximum allele-sharing LOD score of 4.81 on chromosome 9q31-q33 was detected. Haplotype analysis identified a region for intracerebral haemorrhage.
Wang *et al.* (2014)^77^	Linkage and association analysis	Ischaemic stroke	227 Chinese families with ischaemic stroke	SNP rs1800798 in the IL-8 gene is signficantly linked to ischaemic stroke (*p* = 0.002) and small arterial occlusion (small-vessel disease) (*p* = 0.022).

## Stroke candidate genes

Identification of the phosphodiesterase 4D (*PDE4D*) and 5-lipoxygenase activating protein (*ALOX5AP*) genes through linkage analysis by the Icelandic Decode group was a significant landmark in the history of stroke genomics.^1^,^2^,^4^ The rs918592 SNP variant of *PDE4D* was found to be significantly associated with stroke in current smokers in an African American cohort,^7^ while mutations in the *NOS3* have also been significantly associated with large-artery stroke in African Americans.^6^ Other variants significantly associated with ischaemic stroke in African Americans include the *IL6R* polymorphisms and the kappacasein gene *CSN3* found on chromosome 4, the latter through exome sequencing.^3^,^5^

The genetics of intracerebral haemorrhage (ICH) has also been explored through a range of candidate gene and GWAS approaches. Genes involved in the renin–angiotensin–aldosterone system, coagulation pathway, lipid metabolism, homocysteine metabolism and inflammation are among the most explored.^83^ The *APOE* ε2 and *APOE* ε4 genes have been associated with lobal ICH in Caucasian, Asian and African American populations with a high prevalence of cerebral amyloid angiopathy (CAA).^84^,^85^

The association of *APOE* ε4 genes with deep ICH is rather inconsistent.^85^ It is widely accepted that CAA is a frequent cause of ICH and the presence of an APOE ε4 allele substantially increases the risk of CAA.^86^ Whether the risk of CAA or ICH is different in Africans is uncertain but it has long been known that the frequency of the APOE ε4 alleles, irrespective of tribal origin, is highly represented in the general African population.^87^

Deep ICH is also more aetiologically related to hypertensive chronic small-vessel disease and is likely to be more relevant in African populations where hypertension is the dominant stroke risk factor. Other genes with significant polymorphisms related to ICH include the methylenetetrahydrofolatereductase (*MTHFR*), interleukin-6, tumour necrosis factor-α, angiotensin converting enzyme (ACE), factor VII, factor XIII, platelet activating factor and β-tubulin, although most are described in populations of non-African ancestry.^83^

## Stroke GWA S and WES studies

The candidate genes approach has proved disappointing in identifying genes contributing to the risk of multifactorial or polygenic stroke. This is a situation shared with other complex diseases.^88^ Recently, the GWAS approach has revolutionised the field of stroke genetics. GWAS enables markers spanning the whole genome to be genotyped in a single experiment. Using a case–control methodology and rigorous statistical methods to account for the multiple comparisons made, associations between completely unexpected chromosomal loci and disease can be identified.^88^,^89^

GWAS has been employed to identify genetic loci for many other cardiovascular diseases such as coronary heart disease, diabetes and hypertension, and is just being applied to stroke. The pitfalls of previous studies of genomic contributions to stroke include poor phenotyping, underpowered studies, confounders, winner’s curse, and non-validation in independent populations.^88^,^90^ For example, the Siblings With Ischemic Stroke study (SWISS) did not demonstrate any significant genome-wide association.^91^ However, certain novel genetic variants have been identified as risk factors in stroke populations, with some being replicated in other populations.

The International Stroke Genetics Consortium and the Wellcome Trust Case–Control Consortium published the largest GWAS for ischaemic stroke carried out to date. This study successfully demonstrated the importance of very large multicentre study samples, identified a new associated genetic variant and replicated findings of previous stroke GWAS. The findings also demonstrated the value of clear phenotyping and the fact that different stroke phenotypes may differ in their genetic architectures. Table 2 summarises the findings of salient recent GWAS studies in stroke, including a single study by Cole *et al.* in 2012, which utilised exome sequencing.^3^,^92^-^103^

**Table 2:** Recent GWA S and WES studies in stroke

*First author*	*Study type*	*Phenotype*	*Sample size*	*Sample ancestry*	*Associated regions*
Hata *et al.* (2011)^85^	GWAS	Ischaemic stroke	1 112 cases, 1 112 controls	Japanese	14q22 (PRKCH), 11q12 (AGTRL1)
Matarin *et al.* (2009)^89^	GWAS	Ischaemic stroke	249 cases, 268 controls	White	None
Gretasrdottri and Gudjartsson *et al.* (2008, 2009)^87^,^88^	GWAS	Ischaemic stroke	1 661 cases, 10 815 controls	Icelandic	4q25 (PITX2), 16q22.3 (ZFHX3)
Bilguvar *et al.* (2008)^89^	GWAS	lntracranial aneurysms	2 100 cases, 8 000 controls	Finish, Dutch, Japanese	2q33 (PLCL1), 8q12 (SOX17), 9p21.3 (CDKN2A, CDK N2B, ANRIL)
lkram *et al.* (2009)^90^	GWAS	Ischaemic stroke	Cohort of 19 602, 1 164 events	Caucasian	12p13.33 (NINJ2)
Yamada *et al.* (2009)^91^	GWAS	Ischaemic stroke	992 cases, 5 349 controls	Japanese	22q13 (CELSR1)
Zhang *et al.* (2012)^92^	GWAS	Ischaemic and haemorrhagic stroke	1 657 cases, 1 664 controls	Chinese	9p21.3 (ANRIL)
Matsushita *et al.* (2010)^93^	GWAS	Atherothrombotic stroke	2 775 cases, 2 839 controls	Japanese	ARHGEF 10
ISGC and WTCCC (2012)^94^	GWAS	Large-vessel stroke	3 548 cases, 5 972 controls	European	7p21.1 (HDAC9); replicated previous finding for cardio-embolic stroke near PITX2 and ZFHX3
Holliday *et al.* (2012)^95^	GWAS	Large-vessel stroke	1 162 cases, 1 244 controls	Australian	6p21.1
Cole *et al.* (2012)^3^	WES	Lacunar stroke	889 cases, 927 controls (10 for exome sequencing)	African American, European American	4q21.1 (CSN3) **identified by exome sequencing following previous GWAS
Zhou *et al.* (2014)^96^	GWAS	Lacunar strokes systemic vasculopathy	9 subjects (exome sequencing)	European American, European	*ADA2* gene

## Genetic studies of stroke in Africa

To date, only a few stroke genetic studies (Table 3) have been reported from North Africa and remarkably, none from sub-Saharan Africa where the burden of stroke is disproportionately heavy and the phenomics of stroke appears relatively different. Saidi and colleagues working consistently with a growing Tunisian stroke cohort have reported significant association between ischaemic stroke and polymorphisms in several genes, including plasminogen activator inhibitor, *APOE* ε4, human plasminogen activator, human platelet antigen, angiotensin converting enzyme *Del/Del* genotype, angiotensinogen, endothelial nitric oxide synthase and aldosterone synthase.^104^-^111^

**Table 3:** Genetic studies of stroke in Africa

*First author (year)*	*Study type*	*Stroke phenotype*	*Sample*	*Salient findings*
Saidi *et al.* (2007)^97^	Genotyping	lschaemic stroke	135 cases, 118 controls (Tunisian)	Altered plasminogen activator inhibitor 1 (PAI-1) and tissue-type plasminogen activator (tPA) levels:
				Significant ↑ in PAI-1 and marked ↓ in tPA levels correlated with 4G/5G, but not with -844G/A, PAI-1 variants
				4G/4G carriers had reduced risk of stroke compared with other genotypes
Saidi *et al.* (2007)^98^	Genotyping	lschaemic stroke	216 cases, 282 controls (Tunisian)	ApoE ε3 lower (0.546 vs 0.736; *p* < 0.001) in stroke vs control
				ApoE ε4 higher (0.370 vs 0.181; *p* < 0.001) in stroke vs control
				Prevalence of Apo ε4-containing phenotypes higher in:
				• ischaemic versus haemorrhagic (*p* < 0.001)
				• small-vessel versus large-vessel stroke cases (*p* < 0.001)
				• increased need for statin drugs (*p* = 0.040).
Mourad *et al.* (2008)^105^	Genotyping	Sickle cell anaemia	20 SCA cases, 10 controls (Egyptian)	Presence or ACE D allele significantly predisposed to stroke in children with sickle cell anaemia (SCA).
Saidi *et al.* (2008)^99^	Genotyping	lschaemic stroke	216 stroke patients, 318 controls (Tunisian)	Human platelet alloantigen (HPA) – 1 a/b (*p* < 0.001) and HPA-5 a/b (*p* < 0.001) alleles were associated with stroke-susceptible genotypes: 1a/b-2a/a-3a/b-4a/a-5a/b protective genotypes: 1a/a-2a/a-3a/a-4a/a-5a/a; 1a/a-2a/a-3a/b-4a/a-5a/a; 1a/b -2a/a-3a/a-4a/a-5a/a; 1a/b-2a/a-3a/b-4a/a-5a/a)
Saidi *et al.* (2008)^100^	Genotyping	lschaemic stroke	329 cases, 444 controls	Lower human platelet alloantigen, HPA-1a (*p* < 0.001) and higher HPA-1b (*p* < 0.001) allele frequencies were seen in cases than control subjects.
				Homozygosity for HPA-1b (*p* < 0.001) alleles was more prevalent in stroke cases than in controls.
Saidi *et al.* (2009)^101^	Genotyping	lschaemic stroke	228 cases, 323 controls	Frequency of APOE ε3 allele and Apo E3/E3 genotype lower (*p* < 0.001) in stroke vs controls
				Frequency of Apo ε4 allele and genotypes (E3/E4 and E4/E4) elevated (*p* < 0.001) in stroke vs controls
				Higher proportion of Apo ε4-carrying + ACE Del/Del positive cases seen in young (< 50 years) patients (*p* = 0.012) and associated with large-vessel stroke (*p* = 0.035).
Saidi *et al.* (2009)^102^	Genotyping	lschaemic stroke	329 cases, 444 controls	Angiotensinogen AGT 174T/235M/-6A, AGT 174T/235T/-6G. AGT 174T/235T/- 6A and AGT 174M/235T/-6A haplotypes were significantly associated with an increased risk of stroke.
Saidi *et al.* (2010)^103^	Genotyping	lschaemic stroke	329 IS patients, 444 controls	Endothelial nitric oxide synthase (eNOS) gene polymorphisms (298Asp allele and 298Asp/4b/-786T and 298Asp/4b/-786C haplotypes, and in addition identified 298Asp/4a/-786T haplotypes) were significantly associated with ischaemic stroke.

A single study from Egypt noted that the presence of the *ACE* D allele significantly predisposed to stroke in children with sickle cell anaemia.^112^ It is, however, significant to note that the people of North Africa have a different ancestral origin (predominantly Arabian and Berber) from sub-Saharan African populations.^9^ Therefore, significant differences may be anticipated in the genomic profile of stroke and subtypes in sub-Saharan Africans.

## Problems and perspectives

Apart from the lack of community-based ideal stroke epidemiological data sets and the challenge of accurate phenotypic characterisation of cases in sub-Saharan Africa, there are other inherent problems of genomic research ranging from the negative impact of cultural and religious beliefs, issues of autonomy of decision making and voluntary participation, as well as poor understanding of the health impact of genomics.^113^-^115^ In a qualitative study assessing knowledge and attitude towards personal genomics testing for complex diseases among Nigerians, even though respondents felt the outcome of genomic testing might aid healthful lifestyle modifications, attitude was influenced by religion and culture, especially aspects that might directly contradict beliefs and practices or lead to actions contradicting religious beliefs.^115^

All these aspects introduce critical ethical issues into the framework of genomics research in Africa, which need to be addressed in order to achieve success and popularise the prospects of personalised genomic medicine. In addition, there are also the challenges of adequate infrastructure for genomic studies and analysis of genomic data, a paucity of appropriately trained scientists and physicians who have the capacity to design, implement and interpret such studies and lead translational applications, and insufficient bio-informaticians with analysis expertise and research managers. Unstable power supply and political instability are other bottlenecks.

## Opportunities through H3 Africa: SIREN charting new paths

Although African populations harbour the greatest human genomic diversity, the potential of this for understanding human evolutionary biology and disparities in health and disease are not yet fully explored. The H3Africa Consortium, with funding support from the National Institutes of Health (NIH) and the Wellcome Trust, is currently executing 24 different disease-based projects involving 50 000 to 75 000 participants across the African continent.^11^ This initiative will deeply enhance our understanding of human genomic variation while unravelling the genomic bases of several communicable and non-communicable diseases on the continent, while facilitating genomic infrastructural development and capacity building.

The H3Africa Consortium is revolutionising genomic research in Africa and closing the huge genomics gap between Africa and the developed world. The initiative will reduce health disparities and enhance understanding of health issues for the benefit of Africans and the human race through the discovery of new genes and disease pathways with therapeutic and preventative potentials.

The Stroke Investigative Research and Education Network (SIREN) project is one of the H3Africa-funded projects. The SIREN investigators propose to explore genomic factors in stroke in 6 000 native West Africans (3 000 case–control pairs) in comparison with 1 000 African Americans (80% of whom are of West African ancestral origin) and 12 000 Americans of European ancestry in the REGARDS study (comparison among three tracks).^116^,^117^ The wide genomic variation of African populations offers a unique opportunity to identify novel genomic variants with causal relationships to stroke across different ethnic groups.

**Fig. 1.**
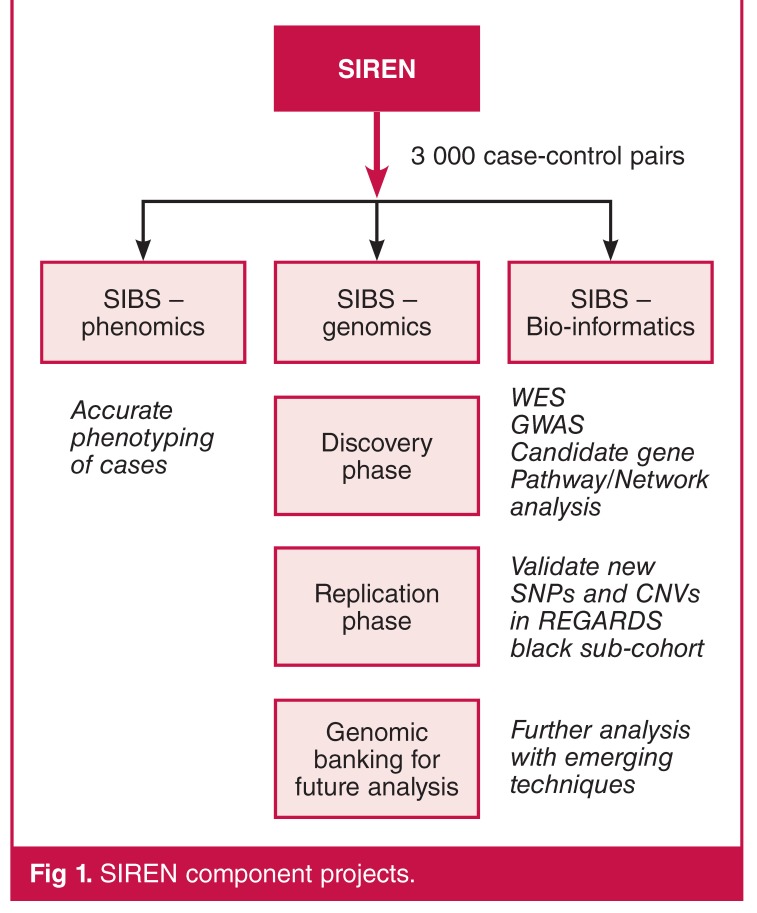
SIREN component projects.

The SIREN project has three main streams: phenomics (including community engagement), genomics, and bio-informatics (Fig. 1). An ethnically diverse sample increases the scope and generalisability of findings, because pan-ethnic replicability of association between a candidate SNP and trait outcome provides support for a causal relationship. However, the high levels of genomic diversity among Africans pose a potential challenge of false-positive associations due to population stratification, while heterogeneity of haplotype structure may reduce statistical power to detect true-positive signals by GWAS.

To combat these challenges, the SIREN project has been designed in compliance with the recommendations of Dichgans *et al.*^88^ with due attention to adequate sample size, rigorous and accurate phenomic characterisation of cases, control for confounders, and planned validation of findings in an independent African American stroke population participating in the REGARDS study^29^,^116^,^117^ (Table 4). In addition, the SIREN project will utilise GWAS approaches using customised chips including unique African variants,^10^ whole-genome and whole-exome sequencing (WGS/WES) and other emergent high-throughput approaches for future analyses. Furthermore, ‘pathway-based analysis’ of genomic data^52^ will chart new paths in our understanding of the molecular trajectories of stroke and unravel new options of stroke diagnostics and therapeutics in the emerging milieu of personalised medicine

**Table 4:** Unique features of SIREN meeting the standard criteria for stroke genomics studies

*Criteria for Stroke Genomics Studies81*	*How met in SIREN – SIBS Genomics*
Venice ‘ A’ rating for sample size	large sample size > 3000 case – control pairs
Phenomic characterization	Rigorous phenotypic assessment in patients and in controls
	Detailed investigations (min of CT) and accurate classification using OCSP, TOAST, ASCO, CCS. Data verification and Quality control System
Control for confounders	Measurement and documentation of conventional vascular risk factors to be controlled for in the analysis
External validation	External Validation in REGARDS cohort (12,500)
Others	Low genotyping error rate (Hardy-Weinberg Equilibrium will be stated in cases and controls) Genomic controls, and other methods to account for population stratification, low P value (corrected for multiple testing)

## Conclusion

Understanding the interaction between genetic and environmental conditions that predispose to stroke and impede favourable post-stroke outcomes is crucial for the formulation of targeted treatment strategies aimed at the successful prevention of and recovery from stroke. Unravelling the genomic underpinnings of stroke in populations of African ancestry will greatly improve our broad understanding of the molecular pathways of stroke and likely add substantially to ongoing efforts to mitigate the devastating global consequences of stroke.

The negative impact of cultural and religious beliefs, issues of autonomy of decision making and voluntary participation, as well as poor understanding of the health impact of genomics are potential challenges to translating genomic advances into real-world clinical applications in Africa. These suggest that caution should be exercised with regard to the expectations from stroke genomics research in Africa, while rigorous detection, evaluation, treatment and control of high blood pressure cannot be overemphasised as a pragmatic strategy to curtail stroke in Africa.
